# Supplementary material for: A comprehensive study of genetic regulation and disease associations of plasma circulatory microRNAs using population-level data
Source: Genome Biol. 2024 Oct 21;25:276. doi: 10.1186/s13059-024-03420-6 (PMC11492503; doi:10.1186/s13059-024-03420-6)
Supplement: Supplementary file 2 — Additional file 2: Methods S1. Measurement of circulating miRNA levels in The Rotterdam Study. Methods S2. Description of genetic data in the Rotterdam Study. Methods S3. Replication of miR-eQTLs in independent cohorts. Methods S4. Functional annotation of miR-eQTLs. Methods S5. Cross-omics and colocalisation analysis. Methods S6. Phenome-wide association studies. Methods S7. Mendelian randomisation. Fig. S1. Selection of study participants in the Rotterdam Study. Fig. S2. Identification of miR-eQTLs and replication in independent cohorts. Fig. S3. Correlation of effect estimates between discovery and replication of miR-eQTLs. Fig. S4. Regional plot for genomic risk loci in chr 14:100655022-101244293 harbouring cis-miR-eQTLs for 31 miRNAs that are clustered together. Fig. S5. Distribution of heritability estimates for 2,083 miRNAs (a). Correlation of heritability estimates with and without principal components (b). Correlation of heritability estimates and the largest proportion of variation explained by single miR-eQTLs (c). Fig. S6. Selection of participants for PheWAS and MR-PheWAS in the UK Biobank. Fig. S7. Schematic network showing miRNAs and disease groups associations (a). Forest plots for 24 associations in MR-PheWAS with no genome-wide significant trans-miR-eQTLs (b). Fig. S8. Scatter plots for MR-PheWAS and replication MR. Fig. S9. Identifying metabolites acting as potential mediators linking miR-1908-5p and benign neoplasm of colon. [file 13059_2024_3420_MOESM2_ESM.docx]

# **Additional file 2.**

# Supplementary Methods

**Methods S1.** Measurement of circulating miRNA levels in The Rotterdam Study

**Methods S2.** Description of genetic data in the Rotterdam Study

**Methods S3.** Replication of miR-eQTLs in independent cohorts

**Methods S4.** Functional annotation of miR-eQTLs

**Methods S5.** Cross-omics and colocalisation analysis

**Methods S6.** Phenome-wide association studies

**Methods S7.** Mendelian randomisation

# Supplementary Figures

**Fig. S1.** Selection of study participants in the Rotterdam Study.

**Fig. S2.** Identification of miR-eQTLs and replication in independent cohorts.

**Fig. S3.** Correlation of effect estimates between discovery and replication of miR-eQTLs.

**Fig. S4.** Regional plot for genomic risk loci in chr 14:100655022-101244293 harbouring cis-miR-eQTLs for 31 miRNAs that are clustered together.

**Fig. S5.** Distribution of heritability estimates for 2,083 miRNAs (a). Correlation of heritability estimates with and without principal components (b). Correlation of heritability estimates and the largest proportion of variation explained by single miR-eQTLs (c).

**Fig. S6.** Selection of participants for PheWAS and MR-PheWAS in the UK Biobank.

**Fig. S7.** a. Schematic network showing miRNAs and disease groups associations. b. Forest plots for 24 associations in MR-PheWAS with no genome-wide significant trans-miR-eQTLs.

**Fig. S8.** Scatter plots for MR-PheWAS and replication MR.

**Fig. S9.** Identifying metabolites acting as potential mediators linking miR-1908-5p and benign neoplasm of colon.

# **Supplementary Methods**

## Methods S1. Measurement of circulating miRNAs in The Rotterdam Study

HTG Edgeseq miRNA whole transcriptome assay (WTA) is a next-generation sequencing (NGS) application that measures the expression of 2,083 human miRNAs described in the miRBase v20 database without the need for extracting RNA. Lysis of collected cells in each tube was performed by applying 50μL of HTG Lysis Buffer (HTG Molecular, Tucson, AZ) to the cap and incubating upside down for 30 minutes at room temperature. After 30 minutes the sample was spun down and 2.8μL of Proteinase K (HTG Molecular, Tucson, AZ, USA) was added, and the sample was then incubated for 180 minutes at 50°C. From each prepared sample, 25μL were added per well to a 96-well sample plate. Samples were run on an HTG EdgeSeq Processor using the HTG EdgeSeq miRNA WTA (HTG Molecular, Tucson, AZ, USA). This assay revolves around nuclease protection, where a pre-selected miRNA population is protected with proprietary protection probes, followed by degradation of all nonhybridized probes and non-targeted RNA by S1 nuclease. This step results in a 1:1 stoichiometric ratio of probes to targeted RNA. Following the processor step, samples were individually barcoded (using a 16-cycle PCR reaction to add adapters and molecular barcodes). Barcoded samples were individually purified using AMPure XP beads (Beckman Coulter, Brea, CA, USA) and quantitated using a KAPA Library Quantification kit (KAPA Biosystem, Wilmington, MA, USA). The library was sequenced on an Illumina MiSeq (Illumina, Inc., San Diego, CA) using a V3 150-cycle kit with two index reads. PhiX was spiked into the library at 5%; this spike-in control is standard for Illumina sequencing libraries. Data were returned from the sequencer in the form of demultiplexed FASTQ files, with one file per original well of the assay. The HTG EdgeSeq Parser (HTG Molecular, Tucson, AZ, USA) was used to align the FASTQ files to the probe list to collate the data. Data were provided as data tables of raw, quality control (QC) raw, counts per million (CPM), and median normalized counts. Log2 counts per million (log2(CPM)) standardization was used to transformed counts and adjusted for total reads within a sample. MiRNAs with log2 CPM <1.0 were indicated as not expressed in the samples.

## Methods S2. Description of genetic data in the Rotterdam Study

Blood samples were drawn at baseline and genotyping was performed using the HumanHap550 Duo BeadChip (Illumina, San Diego, California) for RS-I and RS-II and the Global Screening Array (GSAMD-v3) Illumina array for RS-IV. Samples with a call rate below 97.5%, gender mismatches, excess autosomal heterozygosity, duplicates or family relations, and ethnic outliers were excluded. Variants with call rates below 95.0%, failing missingness test, Hardy‐Weinberg equilibrium P<10^−6^, and allele frequency below 1% were removed. Genotypes were imputed using the MaCH/minimac software to the 1000 Genomes phase I version 3 reference panel or phase 3 version 5 reference panels (for RS-IV). Genetic variants with minor allele frequency < 0.05 and imputation quality < 0.7 were filtered out after genotype imputation.

## Methods S3. Replication of miR-eQTLs in independent cohorts

We used the genome-wide threshold of P<5×10^−08^ and Bonferroni-corrected for 2,083 miRNAs (P<2.4x10^-11^) to identify significant associations. Associations reaching significance in the Rotterdam Study were taken forward for replication in a published miR-eQTLs study by Nikpay et al. (1) using the SNPs or their proxy SNPs (r^2^>0.7 within 500kb on either side of lead SNP position) obtained using LDlinkR (3). Linkage disequilibrium (LD) pruning was used to identify the number of independent SNPs for each miRNA (r^2^<0.01). Similarly, associations identified in previous GWAS by Nikpay et al. (at P<2.4x10^-11^) (1) and Huan et al. in the Framingham Heart Study (at FDR<0.1) (2) were also tested for replication. The Bonferroni threshold was used for replication (α<0.05/n, where n is the total number of SNP-miRNA pairs after pruning). Replication was defined when the associations between SNP and miRNA were Bonferroni-significant in an independent cohort with a concordant direction of effect.

## Methods S4. Functional annotation of miR-eQTLs

Genomic coordinates of miRNAs were extracted from miRBase v20 (<ftp://mirbase.org/pub/mirbase/20/genomes/has.gff3>) (4). Both SNPs and mature miRNA positions were based on Genome Reference Consortium Human Build 37 (GRCh37). The position of each miR-eQTL was mapped as cis or trans with respect to the miRNA position. SNPs located ±500kb upstream and downstream of the start position of mature miRNAs were identified as cis, and those located more than ±500kb away were identified as trans. When certain miRNAs had several paralogs in the genome, we considered all SNPs located ±500kb either side of these regions in the genome as cis.

The web-based tool Functional Mapping and Annotation (FUMA) was used to annotate miR-eQTLs (5). Independent significant miR-eQTLs were defined as those with P<5×10^−08^ in the discovery GWAS or those replicated in independent cohorts and moderate LD with each other at r^2^<0.6. LD calculation was referenced based on the 1000 Genomes phase 3 panel. These SNPs were further clumped to lead SNPs (r^2^ <0.1). Genomic risk loci were then defined based on the lead SNPs when they overlap with a maximum distance of 250kb between LD blocks. The major histocompatibility complex (MHC) region was excluded using the default region between *MOG* and *COL11A2* genes (5,6).

## Methods S5. Cross-omics and colocalisation analysis

Cross-omics and colocalisation analysis leveraged replicated miR-eQTLs with summary statistics for gene expression (eQTLs), protein-QTLs (pQTLs), metabolite-QTLs (met-QTLs), and complex traits. Summary statistics for genetic variants that influence expression levels of mRNA transcripts (cis and trans-eQTLs) in whole blood (N=31,684) were used to identify miR-eQTLs that affect the expression of other genes (7). Summary statistics for pQTLs were obtained from Sun e al. (<https://www.phpc.cam.ac.uk/ceu/proteins/>) (8) and the SCALLOP consortium available through (<https://zenodo.org/record/2615265/>) (9). We used summary statistics from two common metabolomics platforms, Metabolon and Nightingale, to identify miR-eQTLs affecting metabolic pathways by investigating plasma levels of metabolites. Metabolon covers 529 metabolites (N=7,824) (10), while Nightingale covers 123 metabolites in Kettunen et al. (N>20,000) (11) and 249 metabolites in the UK Biobank (N=115,078) (12)~~.~~ The database for intragenic miRNAs was from <https://bmi.ana.med.uni-muenchen.de/miriad/> (13). GWAS Catalog was downloaded from [https://www.ebi.ac.uk/gwas/](https://www.ebi.ac.uk/gwas/docs/file-downloads) (14).

We considered genomic region extending 200kb on either side of mature miRNA position according to miRBase (4). We applied a Bayesian framework to test for the presence of shared causal variant and calculated posterior probability to declare as evidence of colocalisation (15). Colocalisation method estimates the posterior probabilities (PP) of four hypotheses: H0: neither trait has a genetic association in the tested region, H1: only trait 1 (miRNA) has a genetic association in the tested region, H2: only trait 2 (eQTLs/pQTLs/met-QTLs) has a genetic association in the tested region, H3: both traits are associated but with different causal variants, H4: both traits are associated and share a single causal variant. We used default priors in all colocalisation analyses. We reported any results with PP H4>0.5. We also extracted the variant with the highest SNP PP H4 (posterior probability that the SNP being causal conditional on H4 being true) as the likely shared causal variant.

## Methods S6. Phenome-wide association studies

To investigate associations between genetically determined circulatory miRNA and a wide range of clinical diagnoses, a phenome-wide association study (PheWAS) was performed using hospital episode statistics data in the UK Biobank, a large prospective cohort study with over 500,000 individuals aged 40-69 years old recruited between 2006-2010 (16). In brief, participants with genotype and phenotype data were considered in the analysis. Quality control steps taken in the UK Biobank genotype data has been described elsewhere(17).

Our analysis was restricted to participants who identified themselves as “White”. One from each pair of relatives and withdrawn individuals as of August 2021 were excluded. ICD (ninth and tenth editions) codes from the hospital episode statistics data were aligned into phecodes to identify clinically related phenotypes. The analysis was limited to phecodes with at least 200 cases to allow sufficient power for MR analysis (18). PheWAS was conducted using the PheWAS package in R (19).

For each miRNA, associations for genetic variants residing in 500kb on either side of the miRNA position (cis-SNPs) were extracted. The false discovery rate (FDR) was calculated across all cis-SNPs for each miRNA, where those with FDR<0.1 were selected as cis instruments (18). Trans-SNPs associated with circulatory miRNA at P<5x10^-08^ were added as trans instruments in the sensitivity analysis. Instruments were filtered for F-statistics> 10 to avoid weak instrument bias (20). Linkage disequilibrium (LD) clumping for the instruments was conducted using a threshold of r^2^ < 0.1 and a window of 10,000 kb.

For miRNA with single cis miR-eQTL satisfying the instrument criteria, cis-SNP was used as the proxy for corresponding miRNA in single-variant PheWAS. For miRNAs with multiple independent miR-eQTLs, weighted genetic risk scores (GRS) were computed for individuals in the UK Biobank as the sum score of miRNA-increasing alleles of miR-eQTLs identified in the Rotterdam Study using effect sizes as their weights as implemented using PLINK (36). The weighted GRS was rescaled by subtracting GRS from its mean and dividing by its standard deviation to express the association per-SD of the miRNA-increasing allele.

In the main analysis, GRS for each miRNA (miRNA-GRS) was computed from cis-miR-eQTLs (cis-GRS). Additionally, trans-miR-eQTLs at genome-wide significant (P<5x10^-08^) were added, in an extended analysis, to validate findings from cis-GRS. Multiple logistic regression was performed in the UK Biobank for each miRNA-GRS with adjustment for age, sex, genotyping array, and the first five principal components to account for population stratification. Given each phecode is not independent of the other, the false discovery rate (FDR) was calculated for each miRNA-GRS to account for multiple testing (21).

## Methods S7. Mendelian randomisation

Following PheWAS, two-sample Mendelian randomisation (MR) analysis was conducted to assess the causal relationship between candidate miRNAs and outcomes of interest identified from PheWAS (MR-PheWAS). MR-PheWAS considered miRNAs with three or more independent instruments to enable performing robust MR methods as sensitivity analysis. The same set of genetic instruments used in PheWAS contributed to the exposure data in MR-PheWAS. The level of each candidate miRNA was rescaled by subtracting the value from its mean and dividing by its standard deviation (SD) to express the association per-SD increase of the miRNA level. The genetic association between the instruments and the outcome was taken from the UK Biobank. Associations that were significant at FDR<0.05 from MR-PheWAS were taken forward for validation using genetic association estimates (outcome data) from large GWAS consortia or by adding genome-wide significant trans-miR-eQTLs in an extended-MR.

The multiplicative random effect inverse variance weighted method (IVW) was used in the main analysis to combine the effect estimates of the genetic instruments assuming all instruments are valid (22). FDR adjustment was calculated for each miRNA based on p-values of MR-IVW since it was considered the most powerful method when all instruments are valid (22). Robust MR methods which allow the inclusion of pleiotropic variants were used as a sensitivity analysis, including weighted median (WM) or MR-Egger (23-25). WM estimate is valid if less than half of the weight of the genetic instrument is free from horizontal pleiotropy. MR-Egger does not force the regression line through an intercept of zero, making it statistically inefficient but provides a causal estimate corrected for directional horizontal pleiotropy. A non-null intercept in MR-Egger indicates evidence of pleiotropy (24). The agreement among different MR methods was examined to support a robust estimation of causal effects.

Since a liberal LD threshold (r^2^<0.1) was used for clumping, a further sensitivity analysis was conducted by incorporating the correlation matrix between genetic instruments in the fixed effect IVW method (26). MRPRESSO was used to detect outliers (27) and MR analysis was repeated after excluding outliers. Results for MR analysis using different MR methods were presented as forest plots. For replicated associations, reverse MR was conducted to assess the directionality of associations. Independent genetic instruments for complex traits (r^2^<0.001) were identified from large GWAS consortia for the outcomes of interest. Associations between the genetic instruments for complex traits with candidate miRNA levels were extracted from the Rotterdam Study. For top association identified in our study, we also conducted two-sample MR using GWAS on metabolites in 8,299 participants (28) to identify metabolites acting as potential mediators linking candidate miRNA and complex disorder.

We implemented multivariable MR and mediation analysis to disentangle the effect of miRNA and host gene on the disease risk and further identify potential metabolites mediating the effect of miRNA. In particular, we used multivariable MR framework to 1) assess whether observed effect originated from miRNA and independent of the corresponding host genes, 2) select most likely causal mediators, when multiple candidate mediators were present, and 3) estimate the proportion of mediation. As miRNA is often located intragenic, the presence significant association in multivariable MR after adjusting for genetic effect on the host gene support the role of miRNAs in complex diseases independent of host genes.

The genetic instruments for multivariable MR were defined as independent SNPs being strongly associated (P<1.0x10^-05^) with at least one of the exposures of interest (miRNA/host gene/mediator). When assessing potential mediators linking miRNA and disease, both classic MVMR and Bayesian (MR-BMA) methods (29) were used in complementary. When a mediator was identified, two-step MR was conducted to assess the proportion of mediation (30). The first step was univariable MR to estimate total effect of miRNA on the mediator, followed by the second step to estimate direct effect of metabolite to the disease adjusting for the genetic effect on miRNA. Finally, proportion of mediation and corresponding 95% confidence intervals (CIs) were calculated using product coefficient and delta methods.

# **Supplementary Figures**


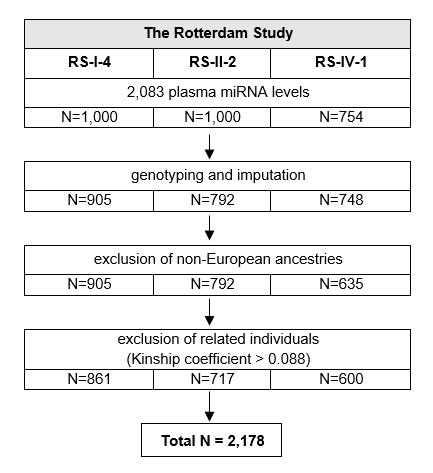


## **Fig. S1.** Selection of study participants in the Rotterdam Study.


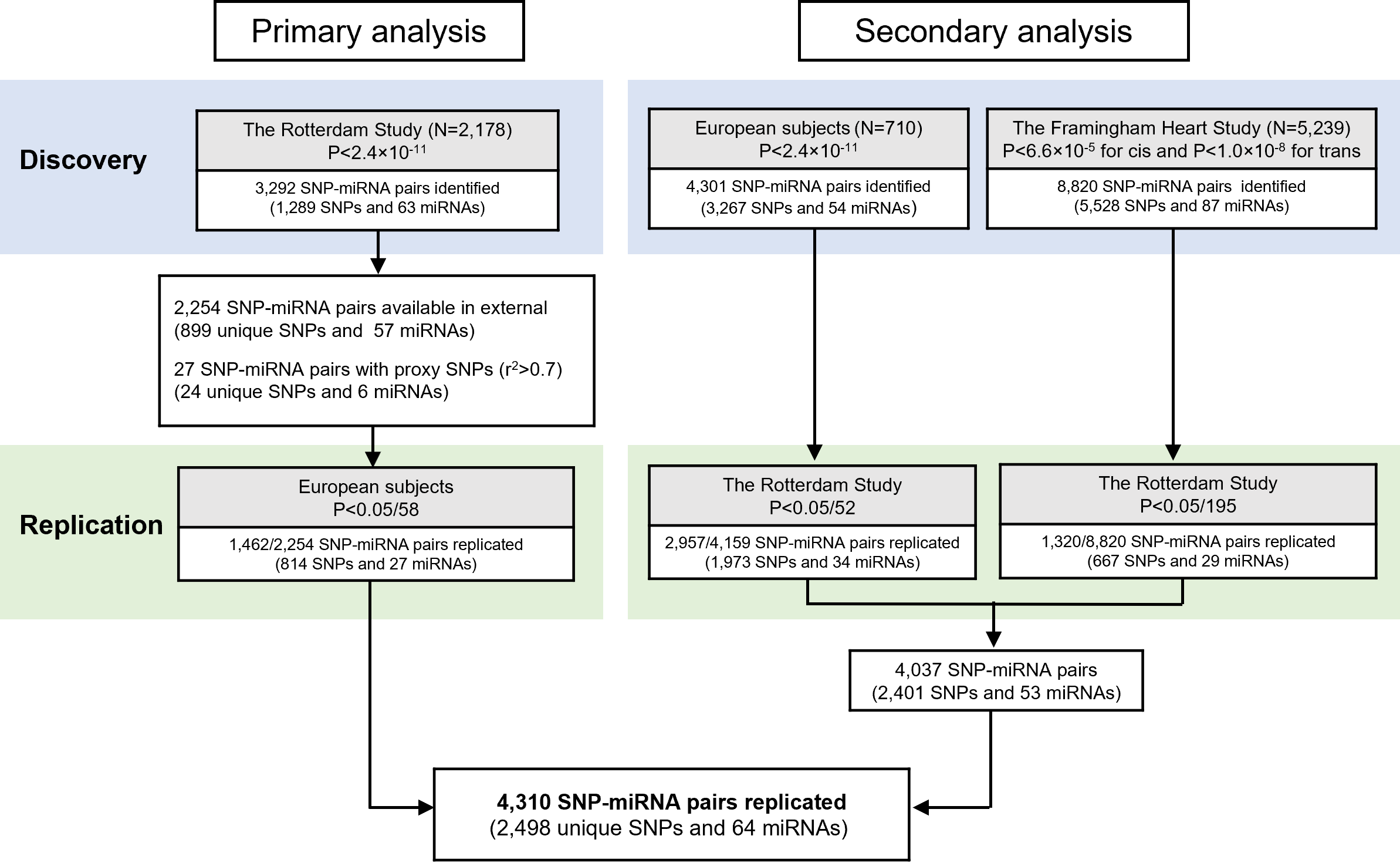


## **Fig. S2.** Identification of miR-eQTLs and replication in independent cohorts. In total, 3,292 significant associations were discovered for 63 miRNAs. Of those 1,462 out of 2,254 associations available in Nikpay et al. (1) were replicated for 27 miRNAs (P<0.05/58). On the other hand, 2,957 associations identified by Nikpay et al. (1) for 34 miRNAs were replicated (P<0.05/52) and 1,320 associations for 29 miRNAs identified in the Framingham Heart Study (2) were also replicated (P<0.05/195). Collectively, 4,310 associations for 64 miRNAs were successfully replicated across studies.

1.
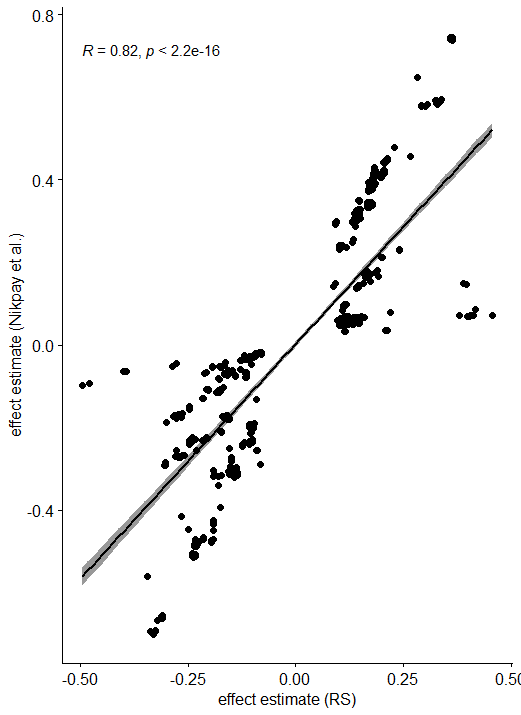


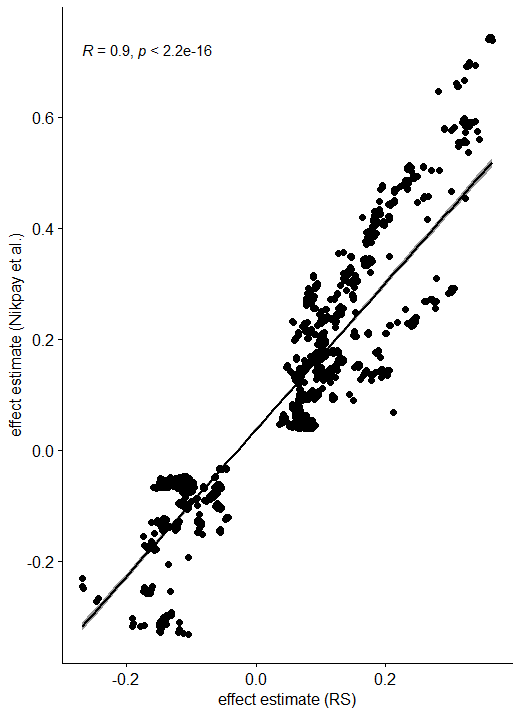
b.

c.


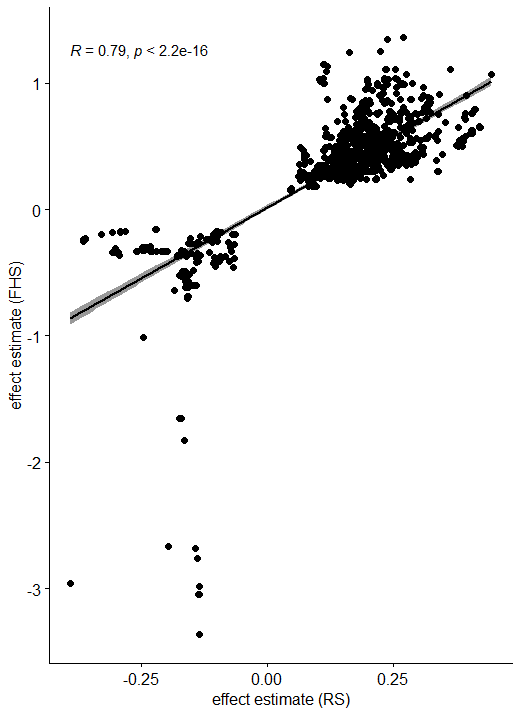


## **Fig. S3**. Correlation of effect estimates between discovery and replication of miR-eQTLs. a. Associations in the Rotterdam Study that were replicated in Nikpay et al.(1). b. Associations in Nikpay et al. (1) that were replicated in the Rotterdam Study. c. Associations in the Framingham Study that were replicated in the Rotterdam Study. For replication, we harmonized the alleles so that the effect estimates between discovery and replication cohorts corresponded to the same effect alleles.


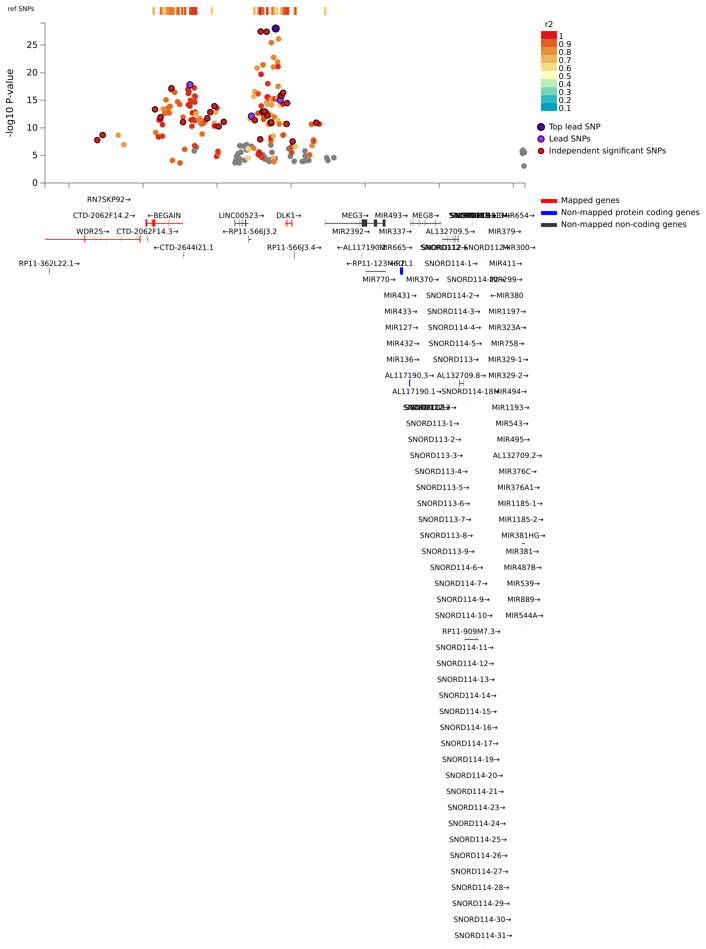


## **Fig. S4.** Regional plot for genomic risk loci in chr 14:100655022-101244293 harbouring cis-miR-eQTLs for 31 miRNAs that are clustered together. Plot was extracted from FUMA.

a.


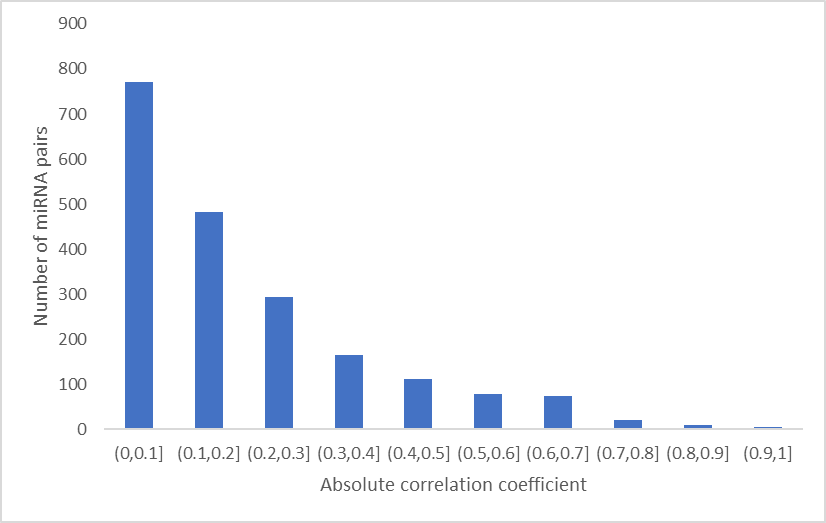


b.


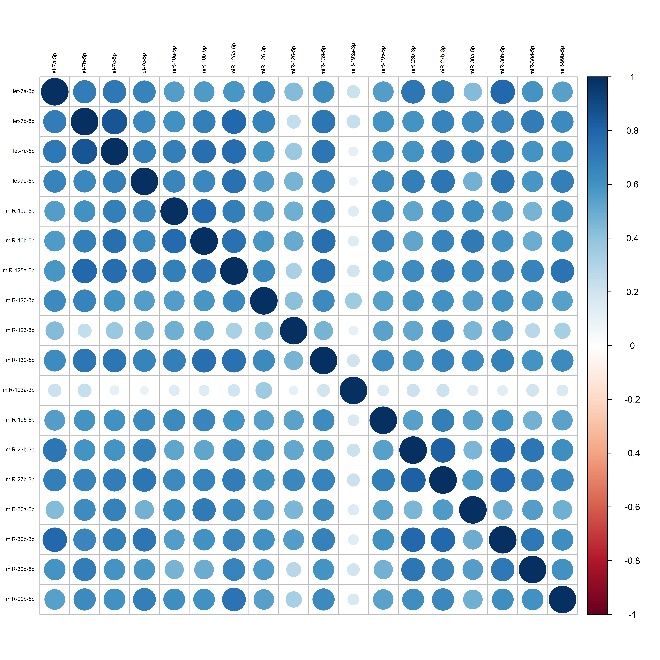
chr14:100655022-101244293 (23 miRNAs) chr9:136128546-136296530 (18 miRNAs)


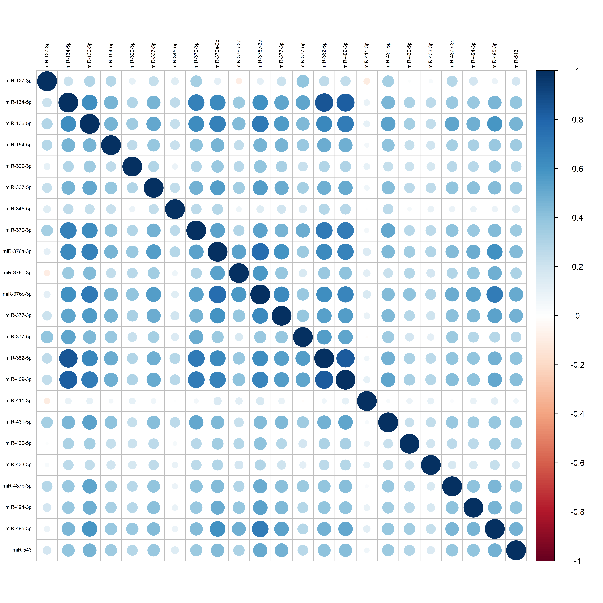


## **Fig. S5.** a. Number of miRNA pairs with genetic findings (64x43 miRNAs pairs tested), categorised by their absolute correlation coefficient. b. Correlation plots between miRNAs within two most pleiotropic loci


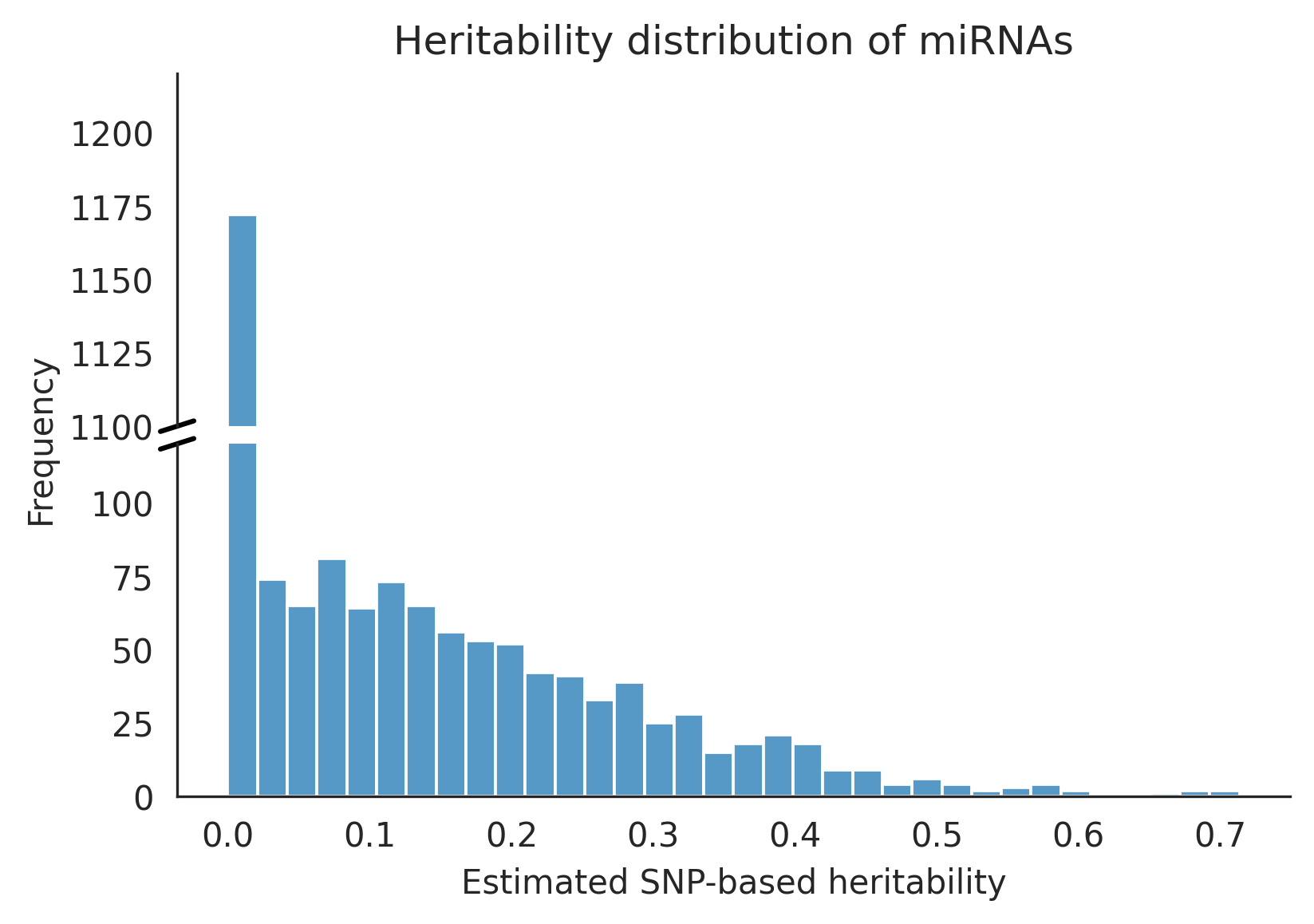
a.

b.


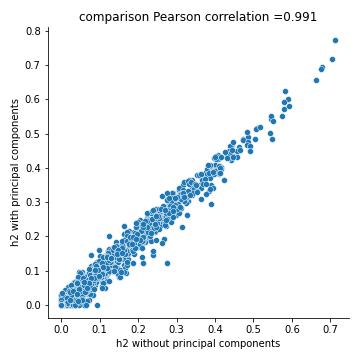


c.


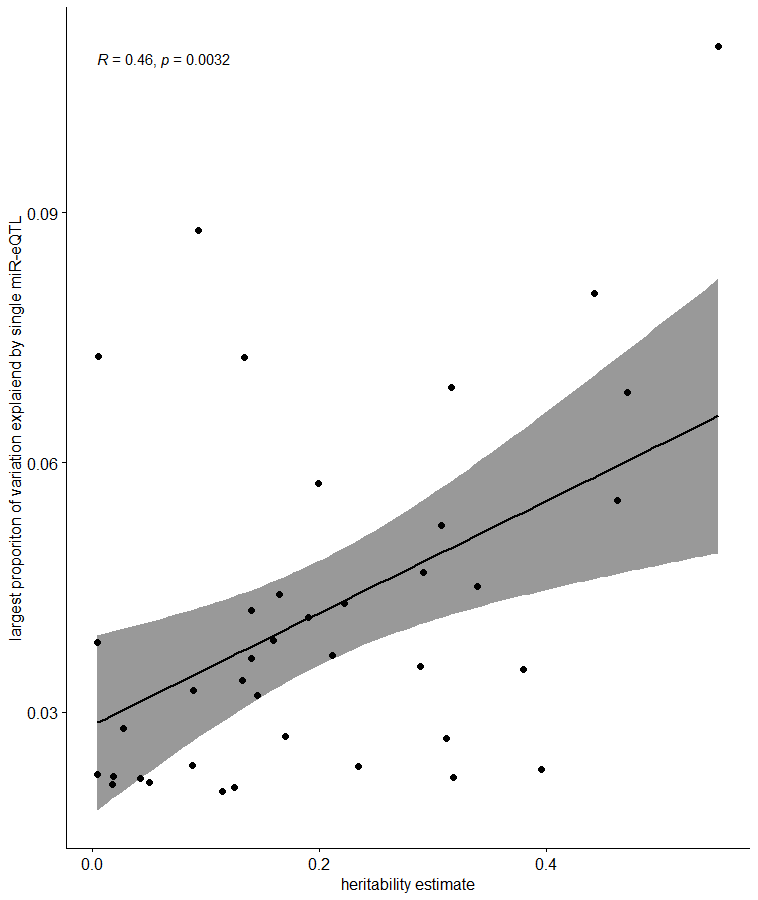


## **Fig. S6**. a. Distribution of heritability estimates for 2,083 miRNAs. b. Pearson correlation of heritability estimates with (y-axis) and without principal components (x-axis). c. Pearson correlation of heritability estimates (x-axis) and the largest proportion of variation explained by single miR-eQTLs. The figure was plotted for miRNAs with non-zero heritability estimates and with at least significant miR-eQTLs.


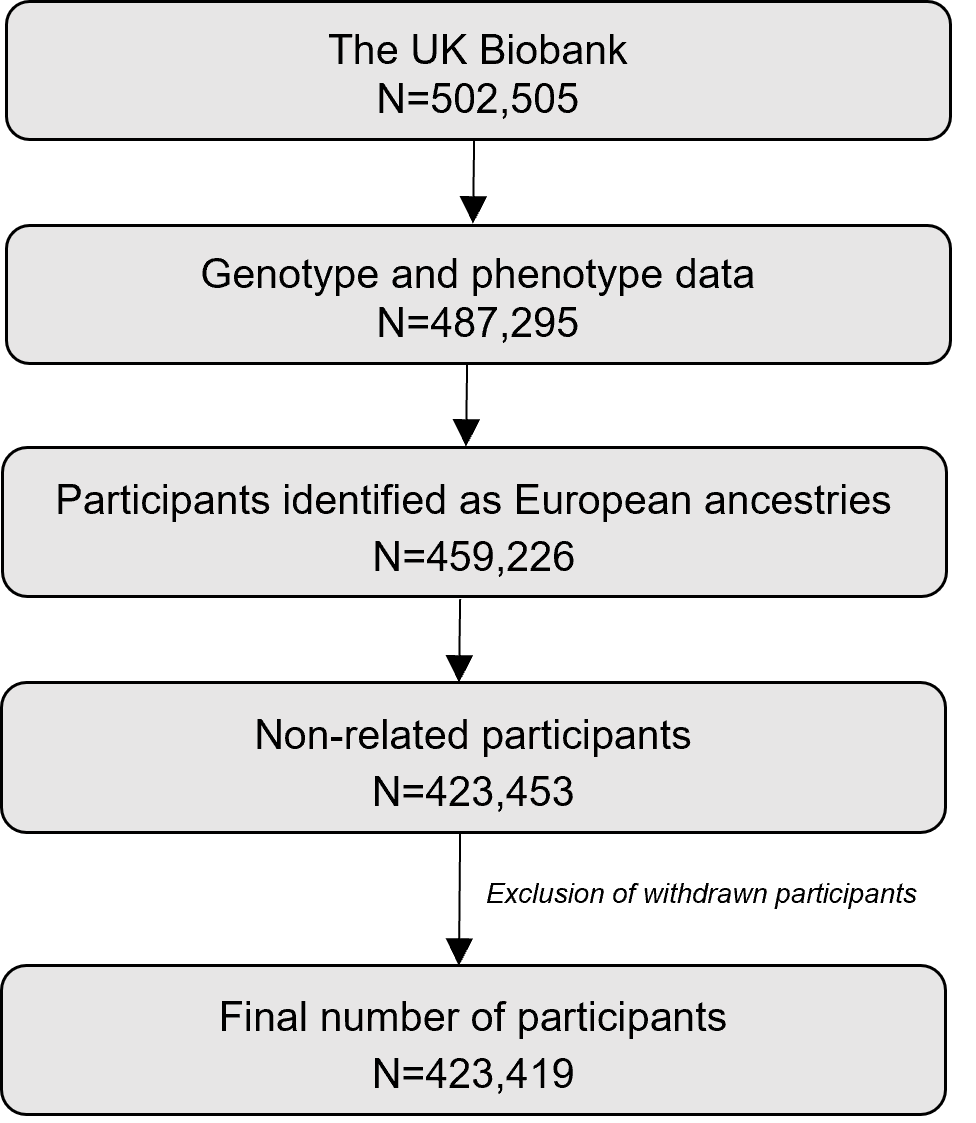


## **Fig. S7.** Selection of participants for PheWAS and MR-PheWAS in the UK Biobank.

1.
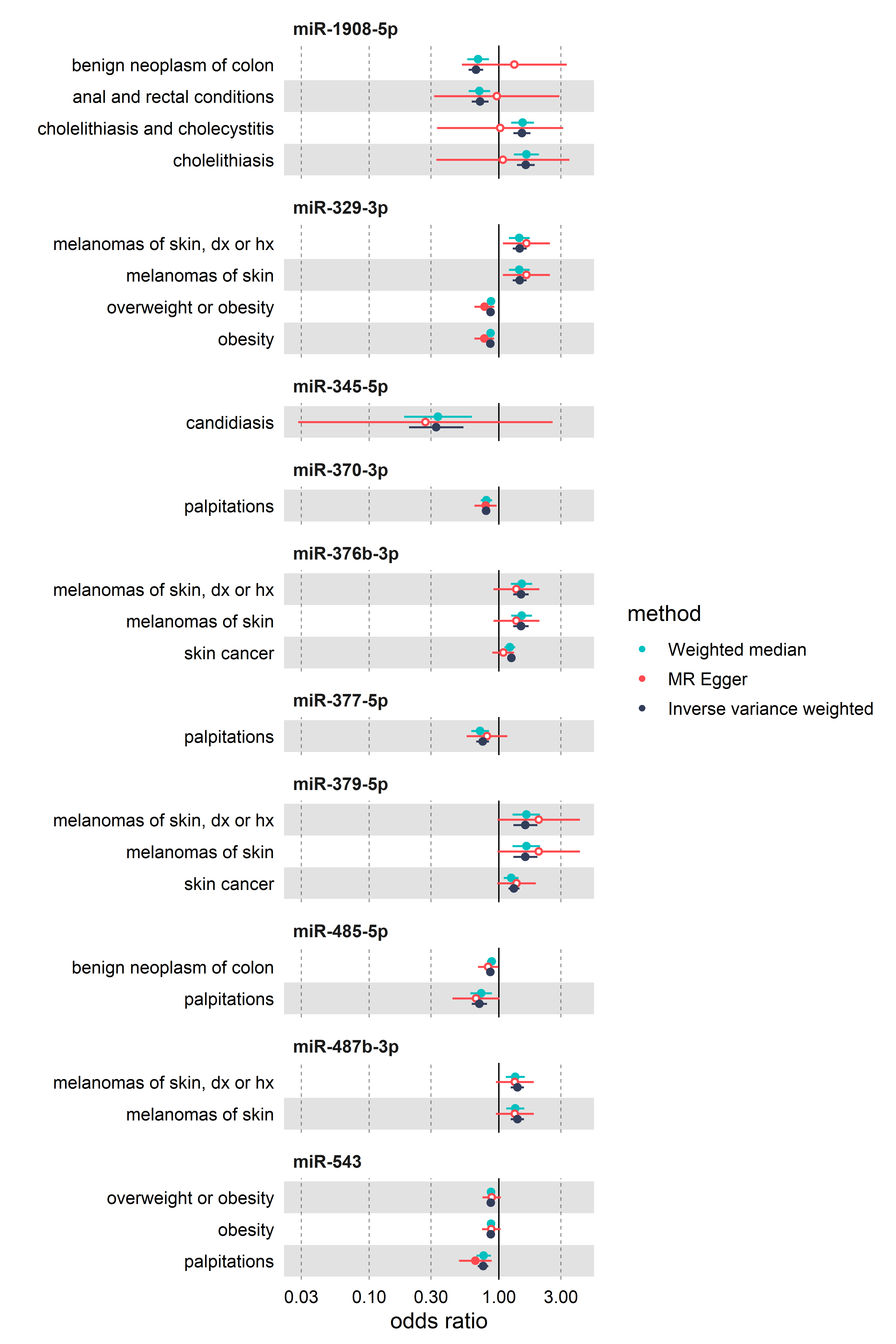

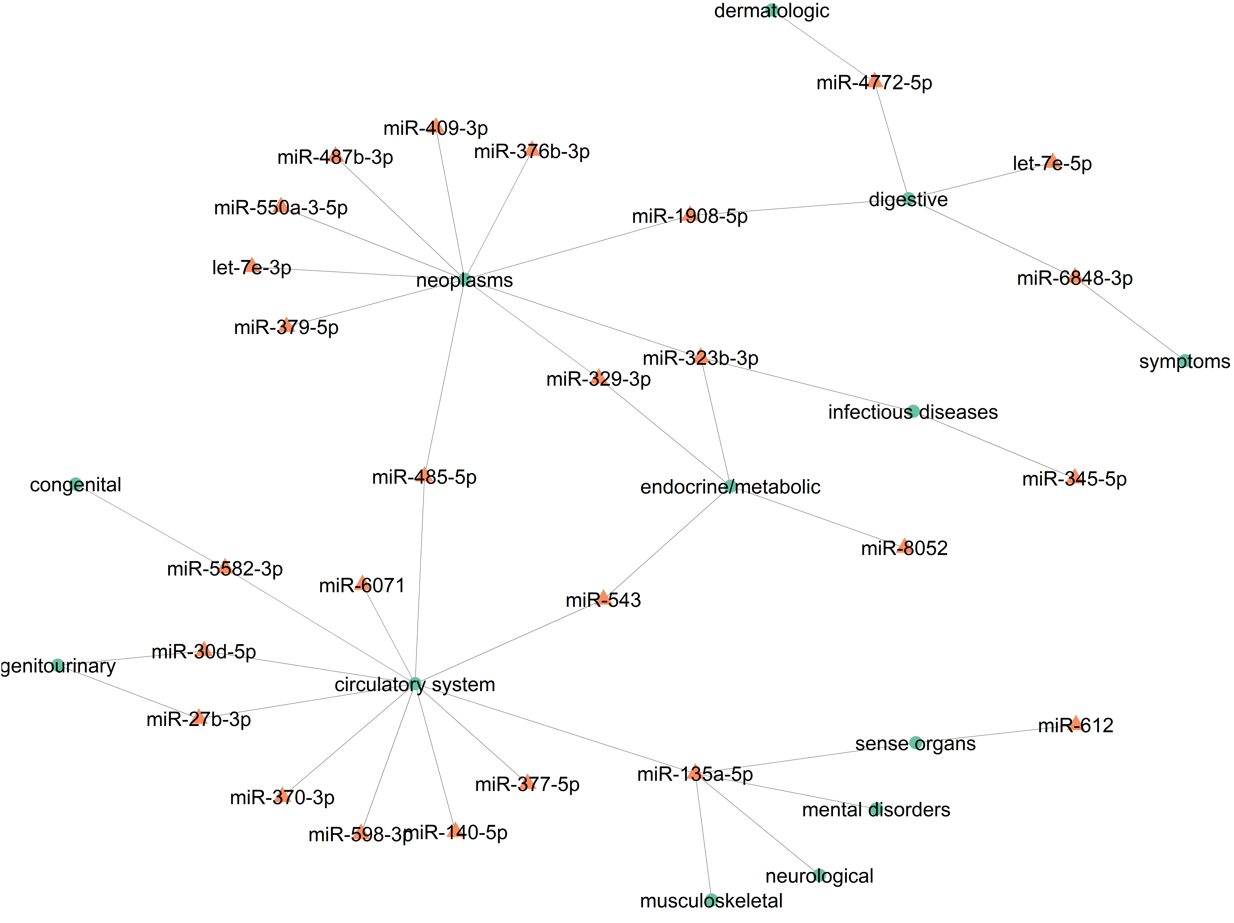
 b.

## **Fig. S8.** **a. Schematic network showing miRNAs and disease groups associations.** Each line corresponds to association between miRNA and clinical diagnosis that belong to a particular disease group. The colour of the circles indicates miRNA (orange) or disease groups (green). **b. Forest plots for 24 associations in MR-PheWAS with no genome-wide significant trans-miR-eQTLs.** Different colours correspond to different MR methods, as labelled.

a.


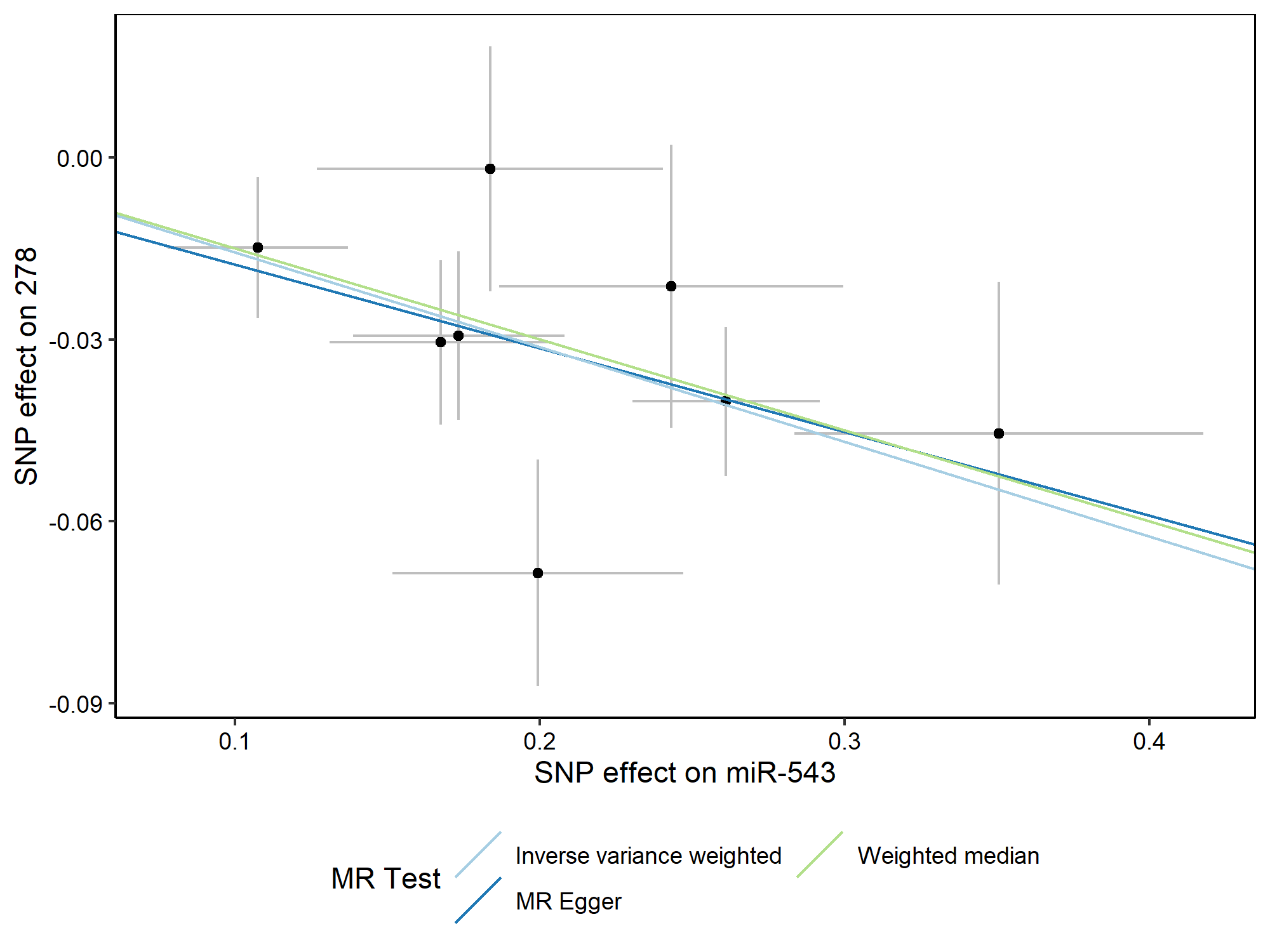

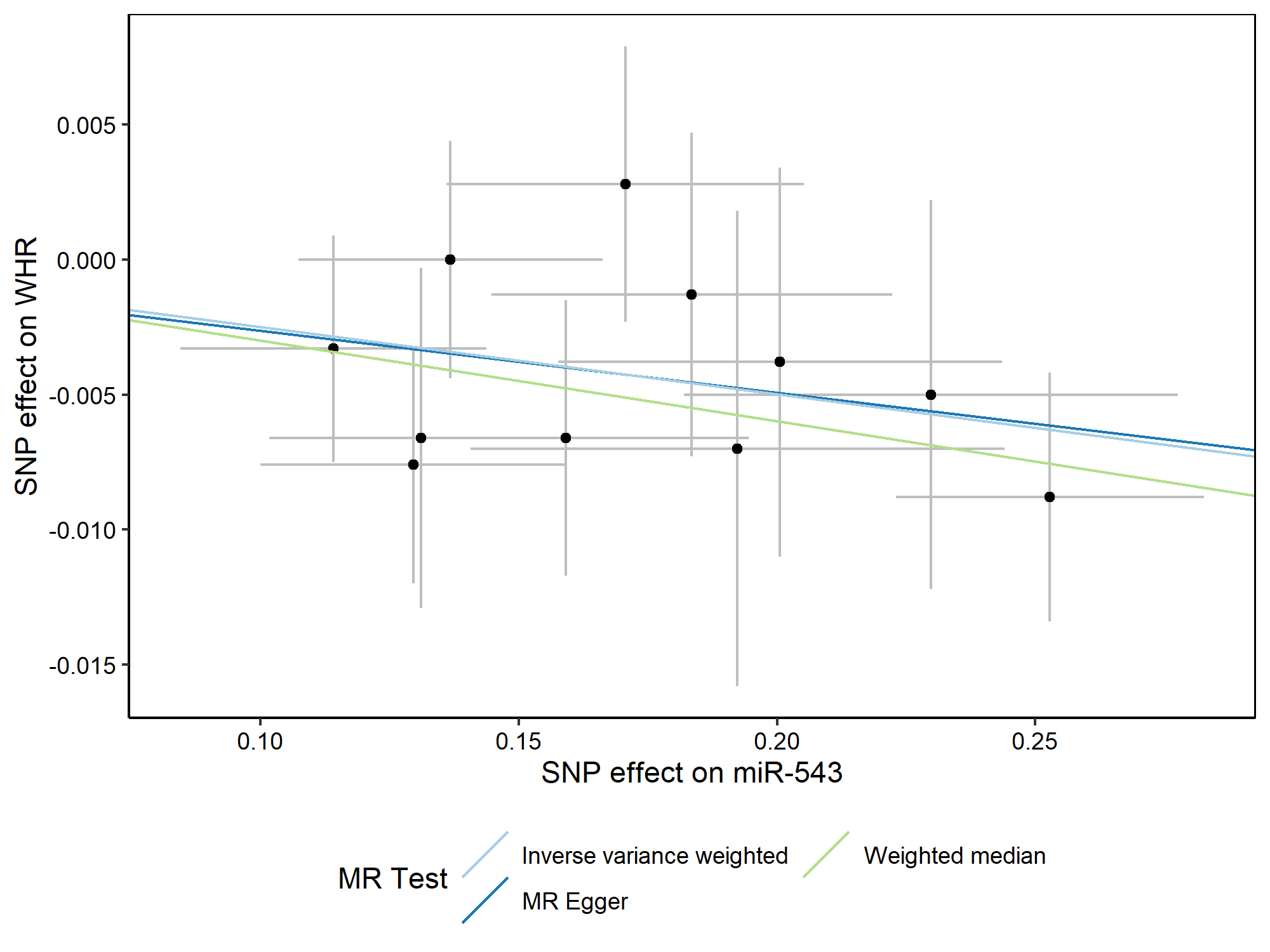


b.


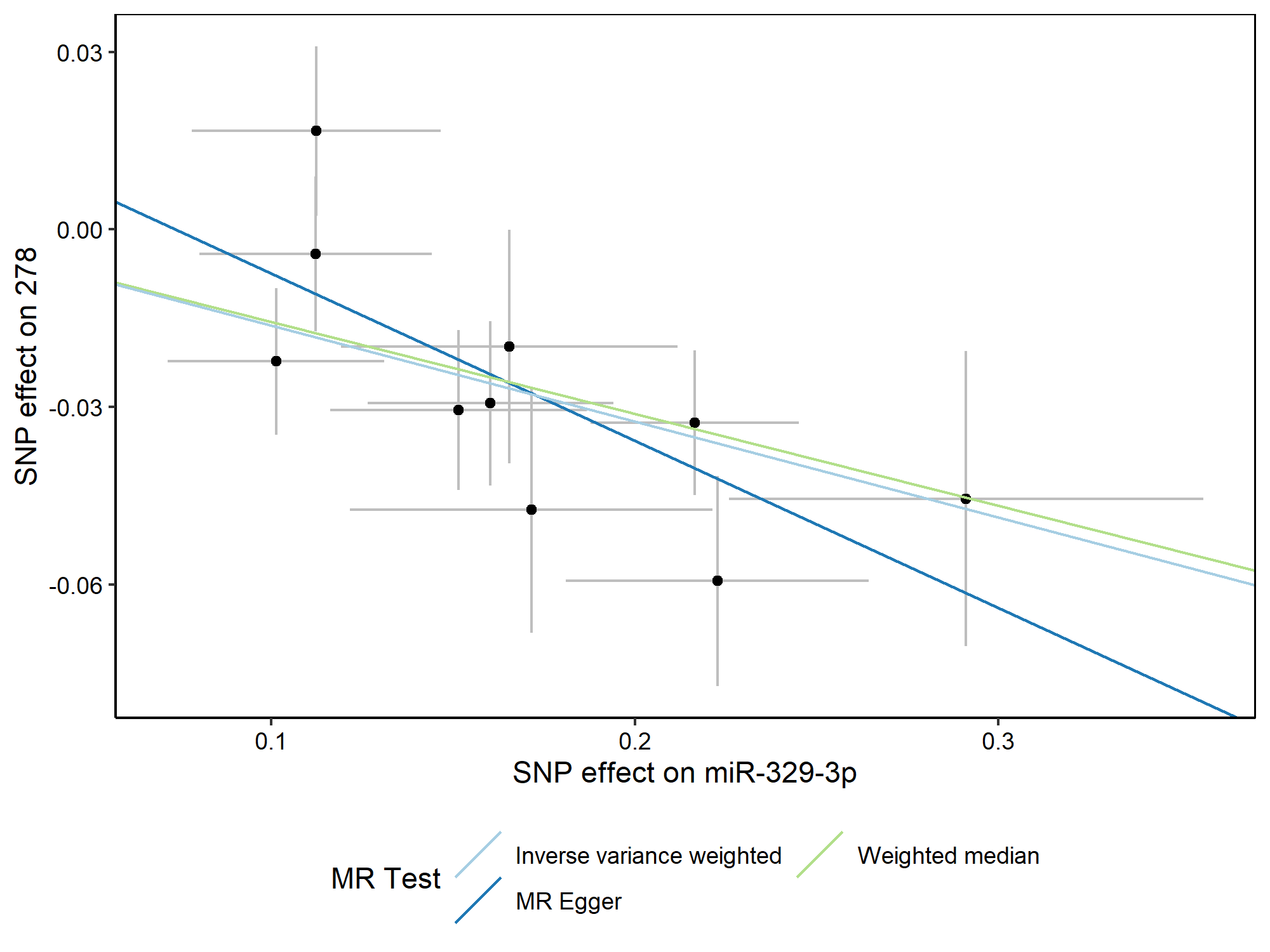

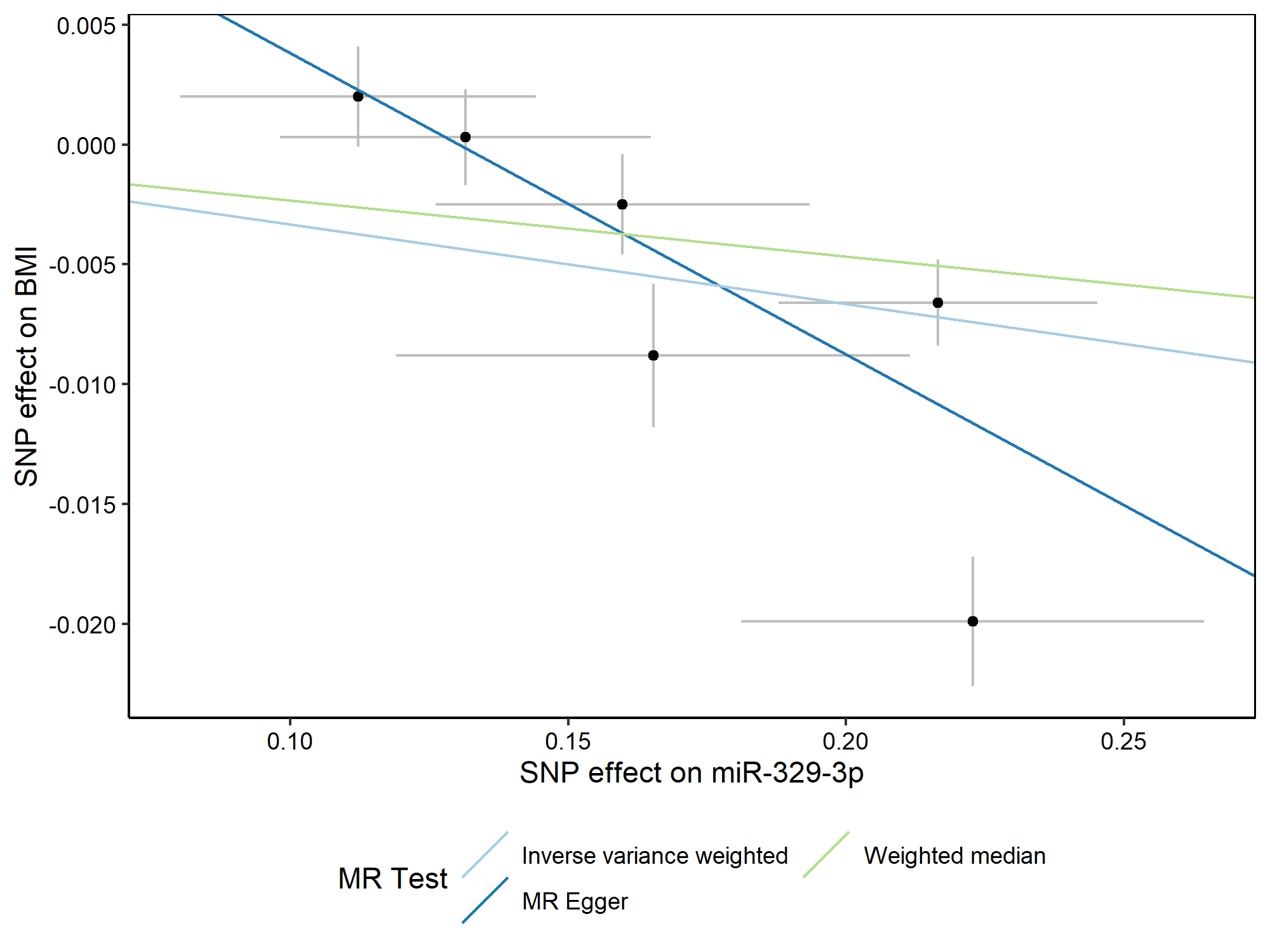


## **Fig. S9.** Scatter plots for MR-PheWAS and replication MR (bottom). a. miR-543 and obesity (left) and waist to hip ratio (right). b. miR-329-3p and obesity (left) and body mass index (right).

a.


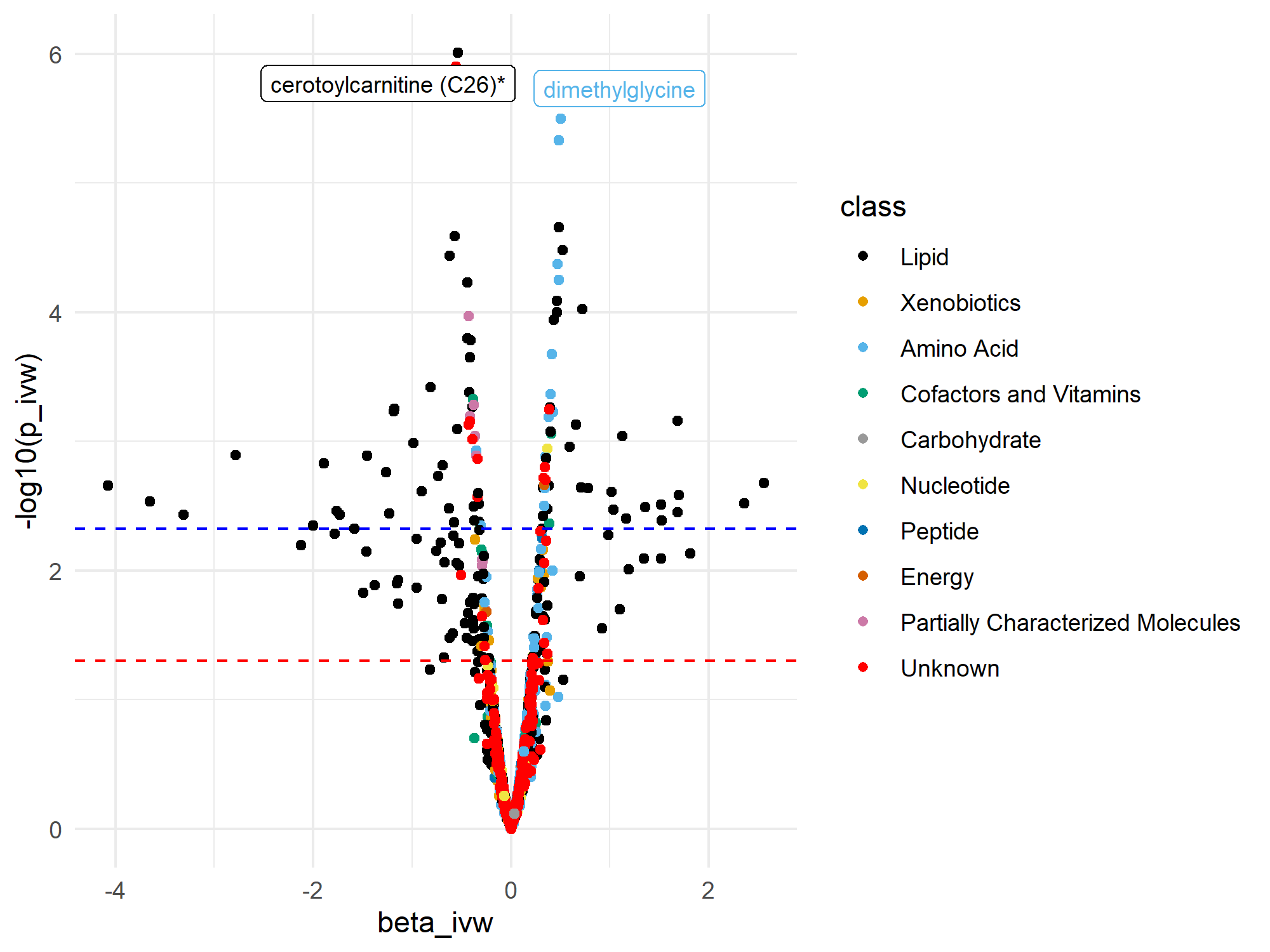


b.


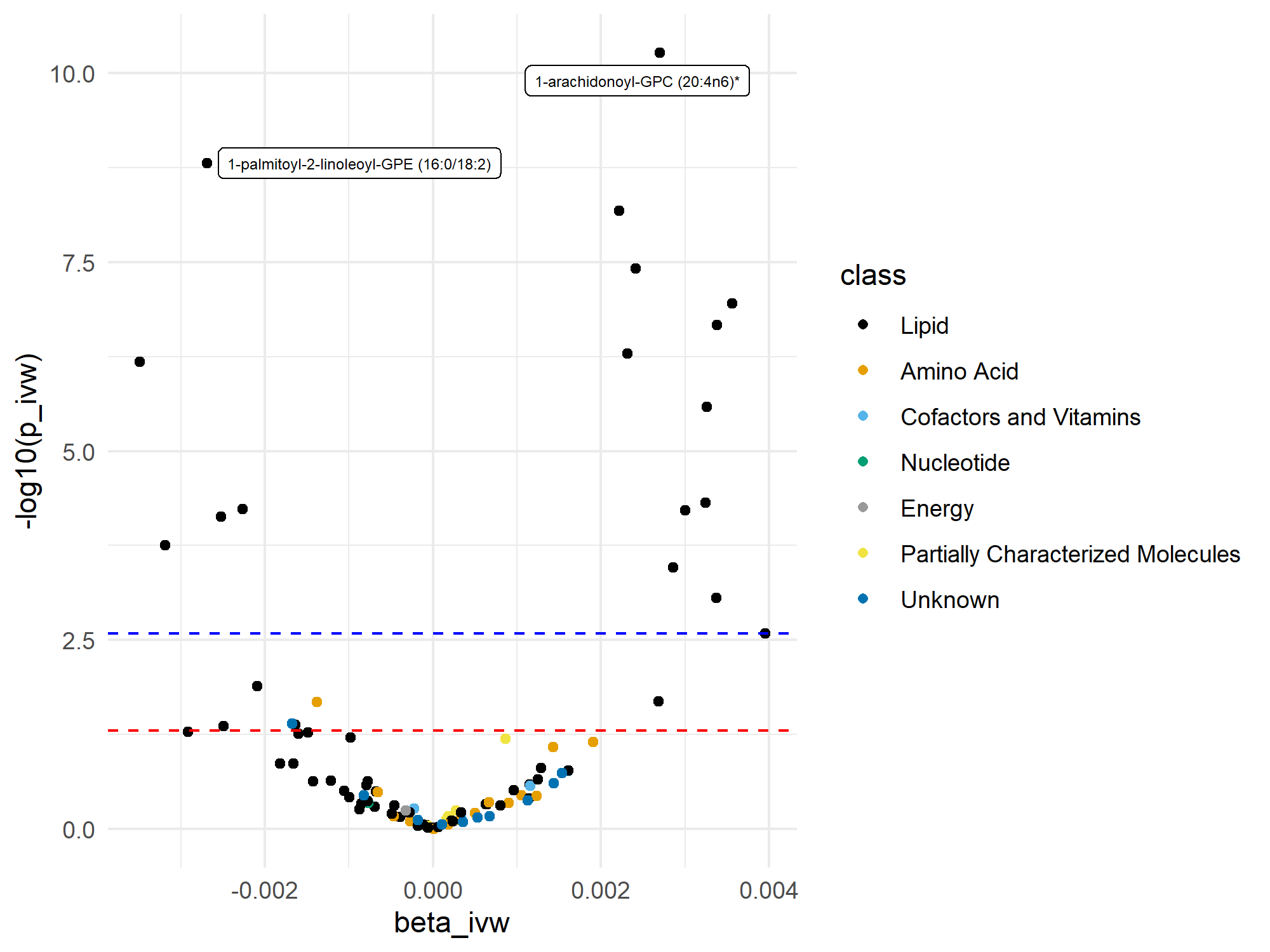


c.


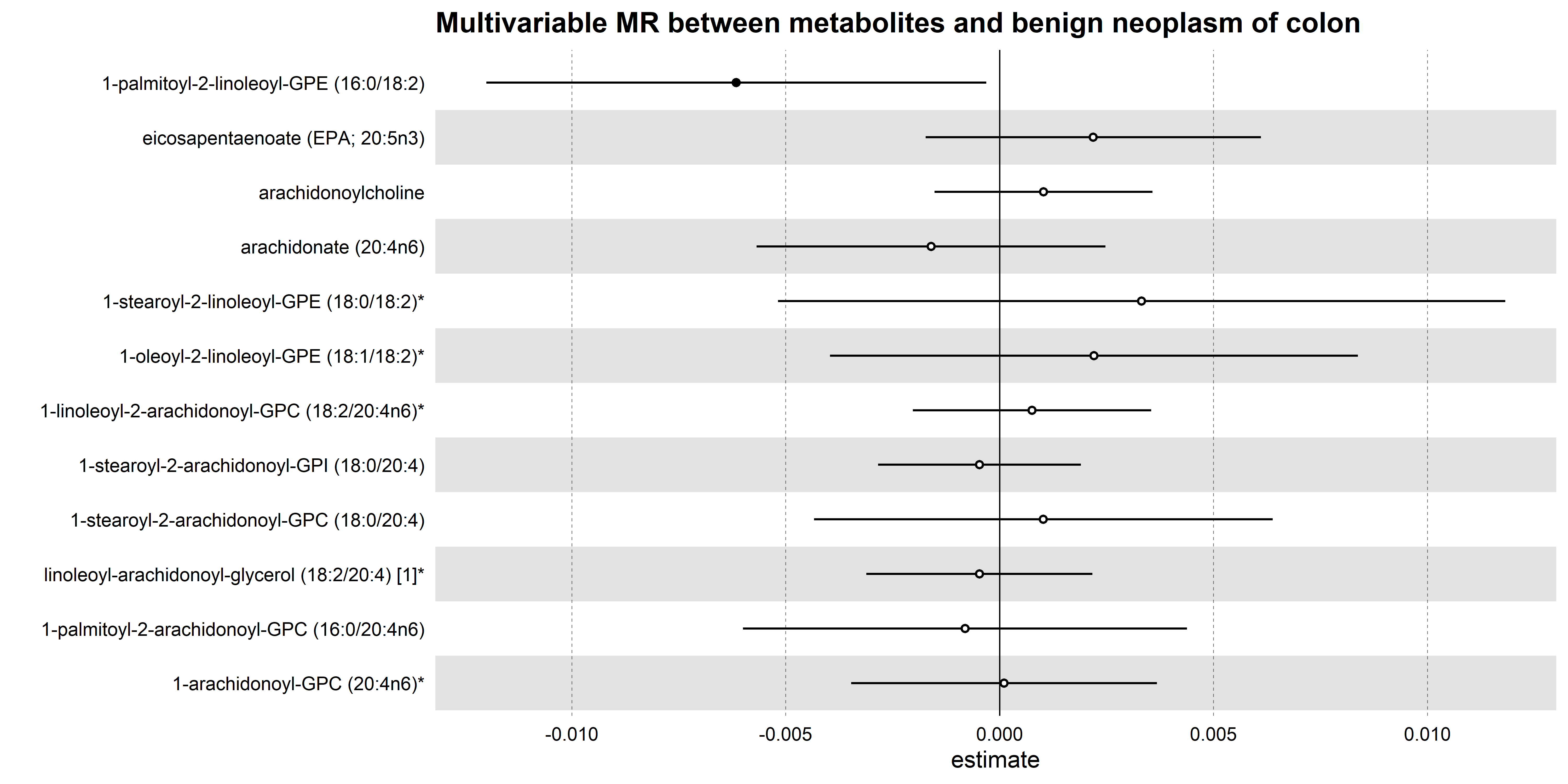


## **Fig. S10. Identifying metabolites acting as potential mediators linking miR-1908-5p and benign neoplasm of colon.** A. Univariable MR analysis between miR-1908-5p and metabolites characterised by Metabolon platform. B. Univariable MR analysis between candidate metabolites and risk of benign neoplasm of colon. C. Multivariable MR analysis between 12 candidate metabolites and risk of benign neoplasm of colon.

# **References**

(1) Nikpay M, Beehler K, Valsesia A, Hager J, Harper M, Dent R, et al. Genome-wide identification of circulating-miRNA expression quantitative trait loci reveals the role of several miRNAs in the regulation of cardiometabolic phenotypes. *Cardiovascular research.* 2019; 115 (11): 1629–1645.

(2) Huan T, Rong J, Liu C, Zhang X, Tanriverdi K, Joehanes R, et al. Genome-wide identification of microRNA expression quantitative trait loci. *Nature communications.* 2015; 6 (1): 1–9.

(3) Machiela MJ, Chanock SJ. LDlink: a web-based application for exploring population-specific haplotype structure and linking correlated alleles of possible functional variants. *Bioinformatics.* 2015; 31 (21): 3555–3557.

(4) Kozomara A, Birgaoanu M, Griffiths-Jones S. miRBase: from microRNA sequences to function. *Nucleic acids research.* 2019; 47 (D1): D155–D162.

(5) Watanabe K, Taskesen E, Van Bochoven A, Posthuma D. Functional mapping and annotation of genetic associations with FUMA. *Nature communications.* 2017; 8 (1): 1–11.

(6) MHC Sequencing Consortium. Complete sequence and gene map of a human major histocompatibility complex. *Nature.* 1999; 401 (6756): 921–923.

(7) Võsa U, Claringbould A, Westra H, Bonder MJ, Deelen P, Zeng B, et al. Large-scale cis-and trans-eQTL analyses identify thousands of genetic loci and polygenic scores that regulate blood gene expression. *Nature genetics.* 2021; 1–11.

(8) Sun BB, Maranville JC, Peters JE, Stacey D, Staley JR, Blackshaw J, et al. Genomic atlas of the human plasma proteome. *Nature.* 2018; 558 (7708): 73–79.

(9) Folkersen L, Gustafsson S, Wang Q, Hansen DH, Hedman ÅK, Schork A, et al. Genomic and drug target evaluation of 90 cardiovascular proteins in 30,931 individuals. *Nature metabolism.* 2020; 2 (10): 1135–1148.

(10) Shin S, Fauman EB, Petersen A, Krumsiek J, Santos R, Huang J, et al. An atlas of genetic influences on human blood metabolites. *Nature genetics.* 2014; 46 (6): 543–550.

(11) Kettunen J, Demirkan A, Würtz P, Draisma HH, Haller T, Rawal R, et al. Genome-wide study for circulating metabolites identifies 62 loci and reveals novel systemic effects of LPA. *Nature communications.* 2016; 7 (1): 1–9.

(12) Elsworth B, Lyon M, Alexander T, Liu Y, Matthews P, Hallett J, et al. The MRC IEU OpenGWAS data infrastructure. *BioRxiv.* 2020; .

(13) Hinske LC, Franca GS, Torres HA, Ohara DT, Lopes-Ramos CM, Heyn J, et al. miRIAD—integrating microRNA inter-and intragenic data. *Database.* 2014; 2014 .

(14) Buniello A, MacArthur JAL, Cerezo M, Harris LW, Hayhurst J, Malangone C, et al. The NHGRI-EBI GWAS Catalog of published genome-wide association studies, targeted arrays and summary statistics 2019. *Nucleic acids research.* 2019; 47 (D1): D1005–D1012.

(15) Giambartolomei C, Vukcevic D, Schadt EE, Franke L, Hingorani AD, Wallace C, et al. Bayesian test for colocalisation between pairs of genetic association studies using summary statistics. *PLoS genetics.* 2014; 10 (5): e1004383.

(16) Sudlow C, Gallacher J, Allen N, Beral V, Burton P, Danesh J, et al. UK biobank: an open access resource for identifying the causes of a wide range of complex diseases of middle and old age. *Plos med.* 2015; 12 (3): e1001779.

(17) Biobank U. Genotyping of 500,000 UK Biobank participants. *Description of sample processing workflow and preparation of DNA for genotyping.* 2015; 11 .

(18) Verma A, Bradford Y, Dudek S, Lucas AM, Verma SS, Pendergrass SA, et al. A simulation study investigating power estimates in phenome-wide association studies. *BMC bioinformatics.* 2018; 19 (1): 120.

(19) Carroll RJ, Bastarache L, Denny JC. R PheWAS: data analysis and plotting tools for phenome-wide association studies in the R environment. *Bioinformatics.* 2014; 30 (16): 2375–2376.

(20) Burgess S, Thompson SG, CRP CHD Genetics Collaboration. Avoiding bias from weak instruments in Mendelian randomization studies. *International journal of epidemiology.* 2011; 40 (3): 755–764.

(21) Benjamini Y, Hochberg Y. Controlling the false discovery rate: a practical and powerful approach to multiple testing. *Journal of the Royal statistical society: series B (Methodological).* 1995; 57 (1): 289–300.

(22) Burgess S, Butterworth A, Thompson SG. Mendelian randomization analysis with multiple genetic variants using summarized data. *Genetic epidemiology.* 2013; 37 (7): 658–665.

(23) Burgess S, Thompson SG. Interpreting findings from Mendelian randomization using the MR-Egger method. *European journal of epidemiology.* 2017; 32 (5): 377–389.

(24) Bowden J, Davey Smith G, Burgess S. Mendelian randomization with invalid instruments: effect estimation and bias detection through Egger regression. *International journal of epidemiology.* 2015; 44 (2): 512–525.

(25) Bowden J, Davey Smith G, Haycock PC, Burgess S. Consistent estimation in Mendelian randomization with some invalid instruments using a weighted median estimator. *Genetic epidemiology.* 2016; 40 (4): 304–314.

(26) Burgess S, Scott RA, Timpson NJ, Davey Smith G, Thompson SG. Using published data in Mendelian randomization: a blueprint for efficient identification of causal risk factors. *European journal of epidemiology.* 2015; 30 (7): 543–552.

(27) Verbanck M, Chen C, Neale B, Do R. Detection of widespread horizontal pleiotropy in causal relationships inferred from Mendelian randomization between complex traits and diseases. *Nature genetics.* 2018; 50 (5): 693–698.

(28) Chen Y, Lu T, Pettersson-Kymmer U, Stewart ID, Butler-Laporte G, Nakanishi T, et al. Genomic atlas of the plasma metabolome prioritizes metabolites implicated in human diseases. *Nature genetics.* 2023; 1–10.

(29) Zuber V, Colijn JM, Klaver C, Burgess S. Selecting likely causal risk factors from high-throughput experiments using multivariable Mendelian randomization. *Nature communications.* 2020; 11 (1): 1–11.

(30) Carter AR, Sanderson E, Hammerton G, Richmond RC, Davey Smith G, Heron J, et al. Mendelian randomisation for mediation analysis: current methods and challenges for implementation. *European journal of epidemiology.* 2021; 36 (5): 465–478.
